# Microplastics Reduce Short-Term Effects of Environmental Contaminants. Part II: Polyethylene Particles Decrease the Effect of Polycyclic Aromatic Hydrocarbons on Microorganisms

**DOI:** 10.3390/ijerph15020287

**Published:** 2018-02-07

**Authors:** Julia Kleinteich, Sven Seidensticker, Nikolaj Marggrander, Christiane Zarfl

**Affiliations:** Center for Applied Geoscience, Eberhard Karls Universität Tübingen, D-72074 Tübingen, Germany; Sven.Seidensticker@uni-tuebingen.de (S.S.); nikolaj.marggrander@googlemail.com (N.M.); christiane.zarfl@uni-tuebingen.de (C.Z.)

**Keywords:** microplastics, vector effect, freshwater sediment, bacteria, polyethylene, polycyclic aromatic hydrocarbons

## Abstract

Microplastic particles in terrestrial and aquatic ecosystems are currently discussed as an emerging persistent organic pollutant and as acting as a vector for hydrophobic chemicals. Microplastic particles may ultimately deposit and accumulate in soil as well as marine and freshwater sediments where they can be harmful to organisms. In this study, we tested the sensitivity of natural freshwater sediment bacterial communities (by genetic fingerprint) to exposure to microplastics (polyethylene, 2 and 20 mg/g sediment) and microplastics loaded with polycyclic aromatic hydrocarbons (PAHs, phenanthrene and anthracene), using a laboratory-based approach. After two weeks of incubation, the bacterial community composition from an unpolluted river section was altered by high concentrations of microplastics, whereas the community downstream of a wastewater treatment plant remained unchanged. Low microplastic concentrations loaded with phenanthrene or anthracene induced a less pronounced response in the sediment communities compared to the same total amount of phenanthrene or anthracene alone. In addition, biodegradation of the PAHs was reduced. This study shows, that microplastic can affect bacterial community composition in unpolluted freshwater sediments. Moreover, the results indicate that microplastics can serve as a vehicle for hydrophobic pollutants but bioavailability of the latter is reduced by the sorption to microplastics.

## 1. Introduction

In recent decades the widespread distribution of microplastics (MPs) in global ecosystems has been reported for marine, freshwater and terrestrial environments [1,2,3,4,5]. In addition to the accumulation of MP in the water column, marine and freshwater sediments are suspected to be a major sink for plastic particles [6]. Chemical and physical weathering of buoyant particles as well as biofilm formation on the particle surface can increase the density of this newly formed agglomeration and thus increase sedimentation [7,8]. Only a few studies have quantified MP loads in freshwater sediments [9,10,11] so far but modelling studies indicate that large amounts of MPs might be at least temporarily stored in river sediments [4,12]. The concentration of these MPs in sediments are usually higher than those in the water column [5].

Microplastics have been shown to have various direct physical effects on different organismal groups in marine and freshwater environments. This includes direct impact on organisms from all trophic levels due to ingestion, accumulation and immobilization [13,14,15,16,17,18], as well as indirect effects through acting as a surface for biofilm growth [7,19]. In soil and marine sediments, uptake of MP by macroorganisms such as annelids, amphipods and polychaetes has been observed [15,20,21,22,23,24,25]. While many studies focus on microplastic uptake by organisms, only little is known about the toxicological responses by sediment organisms. Specifically, the effects on microorganisms have not been investigated so far.

In addition to direct physical effects on organisms and sediment structure, MPs may also change the chemical properties and therefore the habitat conditions in the sediment. Microplastics are known to sorb hydrophobic organic pollutants from the surrounding media [26]. Hydrophobic pollutants such as polycyclic aromatic hydrocarbons (PAHs) are especially well known for sorbing to various types of natural and anthropogenic particles including sediment particles, organic matter and MPs [27,28,29]. Polycyclic aromatic hydrocarbons stem mostly from natural and anthropogenic combustion processes and include compounds such as phenanthrene and anthracene. It has been shown that PAHs may have negative impacts on several organisms due to their bioaccumulation and high mutagenic potential [27,30,31]. Several PAHs, e.g., anthracene, are considered to be substances of very high concern (SVHC) since they are either classified as PBT (persistent, bioaccumulative, toxic) or CMR (carcinogenic, mutagenic, toxic for reproduction) compounds according to the European Chemicals Legislation “Registration, Evaluation, Authorisation & restriction of Chemicals” (REACH). Polycyclic aromatic hydrocarbons sorb to or are released from plastic particles [32], depending on the concentration gradient and the chemical properties of the pollutant. In consequence, pollutants may sorb to MPs in highly polluted media and may be released in surroundings with lower pollutant concentrations. Chemical compounds from harbours or wastewater treatment plants may, by this process, be transported to less polluted sites including the sediments of fresh- or marine waters. Nevertheless, their vector function in terms of being a relevant uptake pathway for pollutants into organisms is questionable [33,34,35]. This is due to the fact that the total mass of MP particles, as well as chemicals transported by MPs in the environment, is small compared to the natural background presence of these compounds either in dilution or sorbed to organic matter, black carbon, or colloids. Since aquatic organisms would already be in equilibrium with the surrounding media, uptake via microplastic particles should be negligible (Rehse et al., this issue [36]).

Microplastics and translocated pollutants deposited in marine and freshwater sediments may affect organisms living in these habitats. Prokaryotic microorganisms play an integral role in freshwater sediments, e.g., as decomposers of organic matter, in nutrient re-cycling, or degradation of organic pollutants [37,38]. Sediment microbial communities are highly diverse and can adapt quickly to environmental fluctuations [38,39]. Microorganisms can therefore serve as sensitive and fast-responding markers of environmental pollution [40,41,42] and may also respond to contamination with MP particles as well as chemical pollutants.

It has been shown that bacteria can degrade chemical pollutants including PAHs [30] but also that these compounds may have negative effects on certain types of freshwater microorganisms [41]. It is assumed that only the freely dissolved part of PAHs is bioavailable and therefore degraded [43]. In the case of sediments, the freely dissolved PAHs correspond to the amount in the water column and in the pore water. The bioavailability of PAHs is therefore strongly dependent on the sediment composition and the types of carbonaceous material [44,45], since this can influence the sorption capacity of the sediment. However, it remains unknown to which extent pollutants sorbed to plastic particles are bioavailable for organismal processes. Since sorbed PAHs may not be accessible for microbial organisms, the question arises whether MP as an additional particulate phase in the sediment may cause significant changes in the availability of PAHs for organisms and if so, whether it may be sink or source. 

We therefore hypothesize that the bioavailability of PAHs, namely anthracene and phenanthrene, is decreased by the presence of microplastics. In this study, we tested if sediment microbial communities from a natural freshwater environment can serve as sensitive markers for MP pollution and examined the vector effect of MP for two PAHs with sediment microorganism community composition as endpoint. 

## 2. Materials and Methods 

**Figure 1:**
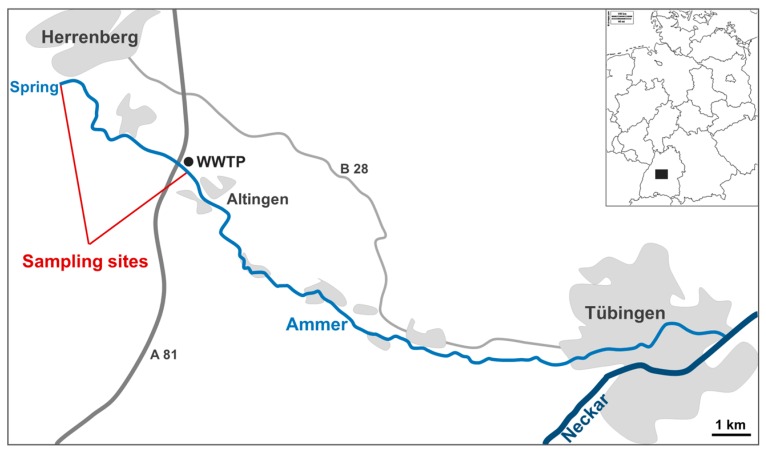
Map of the sampling sites at the River Ammer in southwestern Germany. Urban areas are marked in grey. The location of the wastewater treatment plant (WWTP) for the city of Herrenberg is indicated. Sampling sites are marked in red. For orientation highway A 81 and federal road B 28 are indicated).

Sediment was collected from the river Ammer in southwest Germany (Figure 1) on 11 January 2016 for exposure to MP, on 28 June 2016 for exposure to MP and phenanthrene and on 7 September 2017 for exposure to MP and anthracene. The Ammer is a small river with a mean discharge of 0.96 m^3^/s and a catchment of 165 km^2^, which is dominated by agriculture (71%) and urban areas (17%) [46]. There is an inflow of a wastewater treatment plant ~5 km downstream of the spring, close to the village of Altingen. To compare polluted and unpolluted sites, sediment samples were taken from the river spring (N48.58437°, E8.855240°) as well as from a location 200 m downstream of the wastewater treatment plant effluent (N48.563120°, E8.900621°) on all sampling days.

The sediment was collected in clean and sterilized, brown glass bottles and topped with river water from the corresponding location. Clean and sterilized aluminium foil was placed between the sample and the lid to avoid contact with plastic parts of the lid. The sample bottles were immediately stored at 4 °C in the dark. To avoid long storage, experimental set-up was performed on the following day. River water was decanted to a sterile glass beaker and covered with aluminium foil. From the remaining sediment, pore water was extracted by centrifugation in sterile glass containers (900 rpm, 10 min, Heraeus Megafuge 1. OR from Thermo Fisher Scientific, Waltham, MA, USA). Pore water was then decanted and filtered (0.45 µm). Total organic carbon (TOC) in the sediment and dissolved organic carbon (DOC) in the pore water and the river water were determined by elementar analysers (Elementar Vario TOC cube and Elementar HighTOC, respectively).

For the determination of background concentrations of PAHs, the centrifuged sediment with decreased water content was extracted. First, the sediment was homogenized using a glass spatula. Subsamples were weighed and mixed with clean quartz sand (20/80 *w*/*w*) and filled into metal columns for Accelerated Solvent Extraction (ASE). This was performed in two extraction steps, first with acetone (100 bar, 100 °C) followed by toluene (100 bar, 150 °C). A deuterated internal standard was added (0.2 µg) to the resulting extracts. The acetone extracts were mixed with 800 mL water and 30 mL cyclohexane, followed by mixing for 1 h and phase separation over 24 h. The volume of the resulting cyclohexane was reduced under a gentle stream of nitrogen. The volume of the toluene extract was reduced by rotational evaporation. The concentrations of background PAHs were measured by gas chromatography-mass spectrometry (GC-MS, AgilentG1530A, Santa Clara, CA, USA and Hewlett Packard 5973, mass selective detector, Palo Alto, CA, USA) and quantified via deuterated PAHs as internal standards. The concentrations of PAHs in the water phase were available from Schwientek et al. [46] but were neglected in this study because the concentrations were four orders of magnitude lower than in the sediment.

Standardized plastic particles (Fluorescent Red Polyethylene Microspheres) were purchased from Cospheric LLC (Santa Barbara, CA, USA). The spherical particles had an average size of 212–250 µm, a density of 1.20 g/cm^3^ and contained an orange and fluorescent dye to improve visibility in the experimental setup. Phenanthrene and anthracene were purchased from Sigma-Aldrich Supelco (Bellefonte, PA, USA) and both were loaded separately onto the polyethylene (PE) particles. Of each compound, 80 µg were incubated with 2 g of PE in a 200 mL methanol-water solution (20/80 *v*/*v*) for 96 h in the dark under constant shaking. Subsequently, the particles were separated using a 75 µm mesh size sieve, washed three times with ultrapure water and were dried under a gentle stream of N_2_. A subsample of the spiked PE was extracted with cyclohexane to measure the initial concentration on the particles using GC-MS. Concentrations on the particles were 30.2 and 29.5 µg/g for phenanthrene and anthracene, respectively, with logarithmic partition coefficients between PE and the methanol-water solution, log *K_PE-MeOH/W_*, of 2.49 and 2.37, respectively.

The effect of MPs without pollutant loading was tested on the bacterial communities of natural river sediment. To test if any additives could leach from the MP material prior to the experiment, subsamples (20 mg each, in triplicates) of the MP particles were incubated in ultrapure water or a methanol-water mix (20/80 *v*/*v*) for 14 days. After the incubation, the solvent was removed and extracted with 1 mL cyclohexane. A GC-MS scan did not reveal any additives to be present in the extracts.

**Figure 2:**
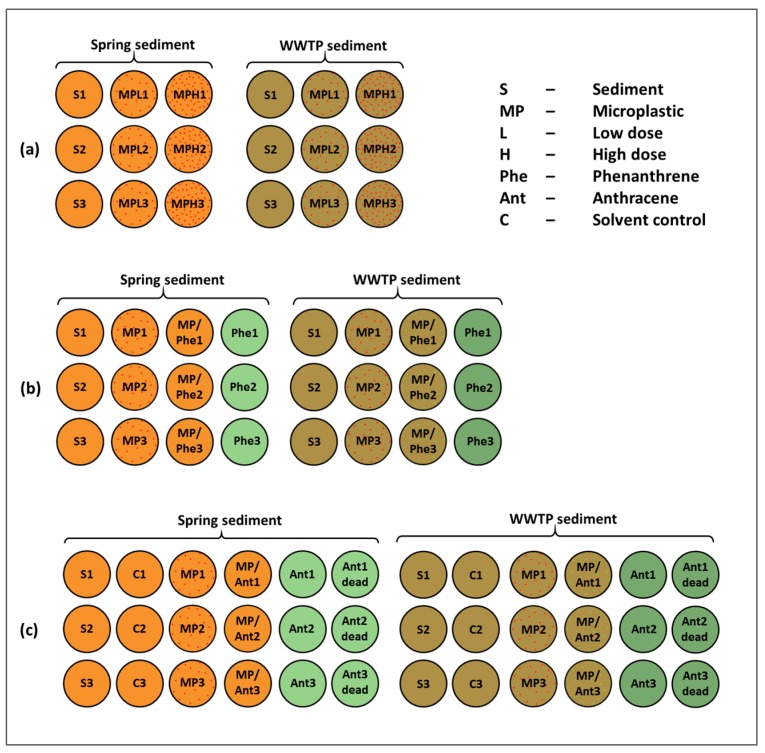
Experimental setup of the three individual experiments with (**a**) microplastic alone, (**b**) phenanthrene and microplastic and (**c**) anthracene and microplastic.

For the setup of the experiment, river sediment was centrifuged as described above for pore water analysis. The supernatant water was decanted to remove excessive liquid and to enhance mixing with microplastic particles. The remaining sediment was homogenized with a sterile glass spatula and 10 g were weighed in sterile glass incubation jars for each experimental replicate. From the same material, subsamples for DNA analysis were taken for day 0 and stored frozen until further use. The sediment was mixed with two different concentrations of MPs, 0.2 g corresponding to ~2570 particles per gram sediment for the high concentration and 0.02 g corresponding to ~257 particles per gram sediment for the low concentration of MPs. Pure sediment without addition of MPs served as negative control. Each treatment group (control, low MP, high MP) was represented by 3 replicates (Figure 2).

After mixing, the sediment-MP-mix was covered with 20 mL of river water from the corresponding sampling site and the incubation jars were covered with a glass lid. The samples were incubated in the dark at 21.8 °C for 14 days. No additional nutrients were added over the whole incubation period. Approximately 0.25 g of sediment was sampled from each jar for DNA analysis and stored frozen in 1.5 mL microcentrifuge tubes until further analysis.

Two co-incubation experiments were performed with PE particles and phenanthrene or anthracene in order to analyse how the presence of MP particles modulates the bioavailability of a PAH with changes in bacterial community composition as an indicator. The exposure experiments were performed similar to the above described experiment with PE particles (Figure 2). In detail, 10 g of river sediment was treated (1) with the lower dose of PE particles (0.02 g per 10 g sediment), or (2) with the same amount of PE particles (0.02 g) loaded with phenanthrene or anthracene (as described above), or (3) without PE particles but with phenanthrene or anthracene according to the amount loaded on the PE particles (0.12 µM phenanthrene in 0.39 mM acetonitrile as solvent or 0.14 µM anthracene). A sediment control without any treatment was applied for both experiments. In the experiment with anthracene, the empty glass jars were pre-treated with an anthracene/acetone solution and the acetone was allowed to vaporize so that no solvent remained in the system [47]. The recovery rate for this method was 92–104%. Nevertheless, we applied a solvent control with the same treatment of acetone in the glass jars. In order to quantify bacterial degradation of anthracene and phenanthrene during the incubation period, we applied an additional control with supplemented sodium azide (NaN_3_, 25 mg) to sterilize and inhibit bacterial degradation.

Each treatment group was represented by three individual replicates and was performed for the two sampling sites of sediment (polluted and unpolluted). As described above, both experiments were run for 14 days at 21 °C in the dark and DNA samples were taken at the end of that period.

To determine the concentration of anthracene and phenanthrene in the remaining sediment and water, the supernatant water was decanted and the sediment and water extracted separately. Water was extracted with cyclohexane and sediment was extracted via accelerated solvent extraction and measured by GC-MS as described above for PAHs. This was not done for the experiment with phenanthrene, so that degradation rates of phenanthrene are estimated based on the natural background of phenanthrene.

The equilibrium fate of pollutants (anthracene and phenanthrene) in the batch system can be calculated according to their partition coefficients. Assuming mass balance, the equilibrium substance concentration *C_w,eq_* in the freely available water phase can be described by:

$$C_{w,eq}=\frac{m_{total}}{V_{w}+K_{OC}\cdot M_{OC}+K_{PE-W}\cdot M_{PE}}$$

whereby *m_total_* is the total substance mass in the experimental system. *V_w_*, *M_OC_* and *M_PE_* are the total volume of water (including pore water), the total mass of organic carbon (in the water column and the sediment) and the mass of PE within the setup, respectively. Equilibrium distribution coefficients log *K_PE-W_* (based on Lohmann [48]) and log *K_OC_* (taken from EPISuite V 4.11, United States Environmental Protection Agency, Washington, DC, USA) were 4.2 and 4.223 for phenanthrene and 4.2 and 4.214 for anthracene, respectively. 

For bacterial fingerprinting, DNA was extracted from the samples taken at day 0 and day 14. Approx. 0.25 g of sediment was extracted using the PowerSoil DNA Isolation Kit (former MO BIO Laboratories, Carlsbad, CA, USA, now Qiagen, Germantown, MD, USA) according to the manufacturer’s instructions with minor modifications. To achieve better cell lysis, samples were incubated at 70 °C for 10 min at 350 rpm (Eppendorf Thermomixer Comfort, Hamburg, Germany). The DNA yield was measured with a NanoDrop^TM^ Spectrophotometer ND-1000 (Thermo Fisher Scientific, Waltham, MA, USA).

Automated Ribosomal Intergenic Spacer Analysis (ARISA) was performed by PCR amplification of the bacterial internal transcribed spacer (ITS) gene region. The primers S-D-Bact-1522-b-S-20 labelled at the 5’ end with the FAM fluorochrome and L-D-Bact-132-a-A-18 (Eurofins Genomics, Ebersberg, Germany). described by Ranjard et al. [49] were used in a final concentration of 500 nM. For PCR amplification, the Q5^®^ High-Fidelity 2× Master Mix (New England Biolabs, Ipswich, MA, USA) was used in 50 µL volumes with 20 ng of DNA per reaction. The PCRs were conducted in a BIO RAD S1000 Thermal Cycler (Hercules, CA, USA) using the following program: 98 °C 60 s [98 °C 10 s, 70 °C 20 s, 72 °C 20 s] × 10, [98 °C 10 s, 70 °C 10 s, 72 °C 20 s] × 25, 72 °C 2 min. PCR Products were cleaned using the New England Biolabs Monarch™ PCR & DNA Cleanup Kit following the manufacturer’s instructions. Yields were measured with the NanoDrop^®^ Spectrophotometer ND-1000 and normalized to 55 ng/μL. Separation of fragments and analysis of peak intensity for the attained ARISA fragment lengths (AFLs) was conducted by Eurofins Genomics via Capillary electrophoresis on an ABI 3130 XL sequencing machine (Applied Biosystems, Foster City, CA, USA) using the LIZ1200 size standard and the ABI-G filter system. Data analysis was performed as described in [50].

For statistical analysis, the number of AFLs is an indicator for the number of bacterial species. Since each bacterial species can have two plasmids with differential genetic coding, species richness is about half of the number of AFLs. The average number of AFLs between different treatments were compared with a two-way ANOVA (sampling site and treatment) and the Bonferroni’s multiple comparisons test using GraphPad Prism 7.03 software (GraphPad Software, Inc., La Jolla, CA, USA). For statistical analysis of AFL data, zero-only rows were removed of the presence-absence matrix and Bray-Curtis similarities calculated using PAST [51]. Non-metric multi dimension scaling graphs (nMDS) were computed using PAST and are based on Bray-Curtis similarities. Analysis of similarities (ANOSIM) was used to calculate differences between treatments and R values using PAST.

## 3. Results

### 3.1. Exposure of Natural River Sediment Bacteria with Microplastics

**Figure 3:**
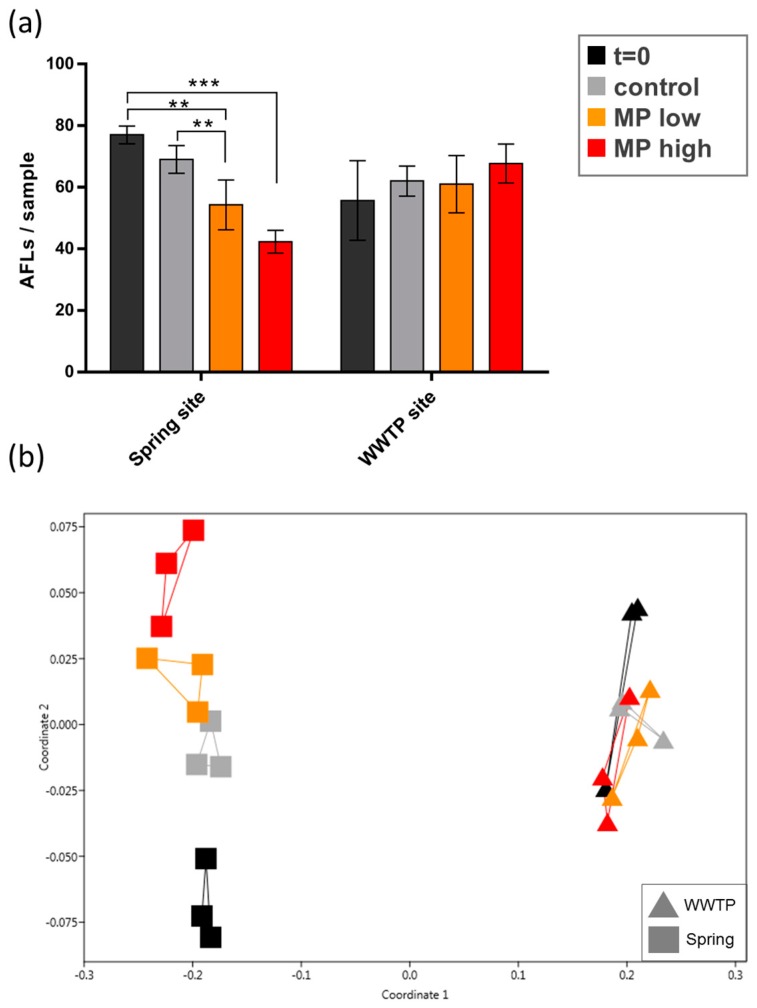
(**a**) Number of Automated Ribosomal Intergenic Spacer Analysis Fragment Length (AFLs) as indicator for bacterial richness in sediments from a spring site and downstream of a wastewater treatment plant (WWTP) before and after treatment with a low (2 mg/g sediment) and high (20 mg/g sediment) dose of PE particles (MP). Significant differences were detected with a two-way ANOVA and Tukey’s multiple comparisons test (** *p* < 0.01, *** *p* < 0.001). (**b**) The data set from (**a**) is represented in a nMDS graph based on Bray-Curtis similarities between samples, showing the shift in bacterial communities before and after treatment with two different doses of PE particles.

The exposure of natural river sediment from a relatively unpolluted site (river spring) with polyethylene (PE) particles showed a dose-response related effect in the species richness (Figure 3a). Both, low and high doses of PE particles induced a significant reduction in the number of AFLs (two-way ANOVA) and therefore the number of bacterial species, when compared to the natural sediment from the spring site. The high dose PE particle exposure but not the low dose, significantly reduced the number of AFLs compared to the non-treated control after 14 days of incubation. In contrast to the unpolluted site, the number of AFLs in sediment collected downstream of the wastewater treatment plant was not affected by the PE particles. This indicates that the bacterial communities at both locations react differently to the MPs. 

The latter was confirmed by nMDS analysis, characterizing the bacterial community composition. Bacterial communities in sediment from the spring and the wastewater treatment plant (WWTP) differed significantly (*p* < 0.001, ANOSIM, Tables S1 and S2) forming two distinctly different clusters in the nMDS graph (Figure 3b). The bacterial community composition from the spring site shifted significantly during the experiment (*p* < 0.001, ANOSIM). High dose MP (R = 0.93, ANOSIM) induced a stronger difference in bacterial communities from the spring site than low dose MP (R = 0.41, ANOSIM) when compared to the control. Low dose and high dose MP showed the smallest difference (R = 0.20, ANOSIM). In contrast, no such shifts were observed for the sediment collected downstream of the WWTP (*p* = 0.40, ANOSIM). In the latter case, neither the experimental conditions, nor the MP particles did have an observable effect on the sediment bacterial composition.

### 3.2. Exposure of Natural River Sediment Bacteria with Microplastics

From the known partition coefficients *K_PE-W_* and *K_OC_*, the equilibrium partitioning of phenanthrene and anthracene was calculated (Table 1). For both locations, the majority of introduced contaminant will partition into the sediment (more than 80%), whereas the second largest fraction will remain in the MP. The distribution patterns for both compounds were similar. The fractions in the water phases are below or equal to 1% and a little higher in the open water compared to the pore water. By calculating the partition coefficients, we could show that sorbed compounds will partly stay within the particles and are therefore not available in biological relevant phases.

**Figure 4:**
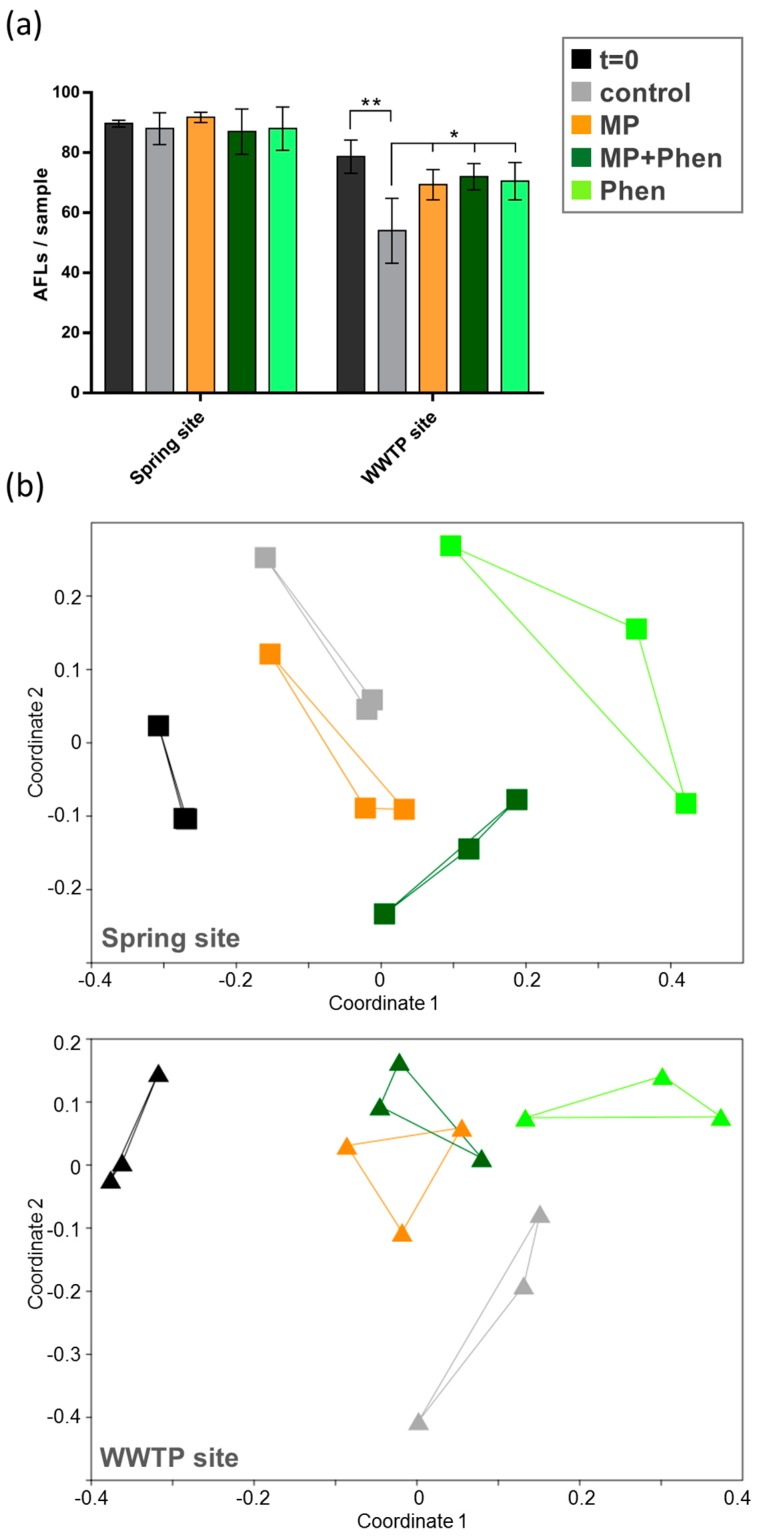
(**a**) Number of Automated Ribosomal Intergenic Spacer Analysis Fragment Length (AFLs) as indicator of bacterial richness in sediments from a spring site and downstream of a wastewater treatment plant (WWTP) before and after treatment with phenanthrene (Phen), polyethylene particles (MP) and phenanthrene-loaded polyethylene particles (MP + Phen). Significant differences were detected with a two-way ANOVA and Tukey’s multiple comparisons test (* *p* < 0.05, ** *p* < 0.01). (**b**) The data set from (**a**) is represented in an nMDS graph based on Bray-Curtis similarities between samples, showing the shift in bacterial communities before and after treatment.

In line with the theoretical distribution of PAHs and hence their bioavailability, the bacterial diversity from both the spring site as well as the site downstream of the WWTP, showed a significant (*p* < 0.01, ANOSIM, Tables S3 and S4) response to the treatment with phenanthrene or PE particles loaded with phenanthrene (Figure 4). At the spring site, the non-treated control group formed a cluster with the low dose PE particle control (R = 0.28, ANOSIM). PE particles loaded with phenanthrene induced a slight shift in the community composition and were separated from the latter cluster. In contrast to PE particles and PE particles loaded with phenanthrene, pure phenanthrene induced a stronger shift in the bacterial community. Both treatments including PE particles were more similar to each other (R = 0.37, ANOSIM) than to the phenanthrene control (R = 0.70 and R = 0.44, ANOSIM). Similar to the experiment with MPs alone, bacterial communities from the spring and the WWTP differed significantly (*p* < 0.001, ANOSIM).

A similar trend was observed for the sediment collected downstream of the WWTP. Here, the bacterial communities exposed to PE particles and PE particles loaded with phenanthrene clustered together (R = 0.09, ANOSIM, Tables S5 and S6), whereas the phenanthrene treatment generated a separate cluster and was different from the other groups (R > 0.68, ANOSIM). The number of bacterial species (AFLs) before and after treatment with phenanthrene and phenanthrene-loaded PE particles was not reduced in sediments from both field sites (Figure 4). Only the control group of the samples from the WWTP showed a significant reduction in number of AFLs (two-way ANOVA and Bonferroni post-test), possibly due to a lack of organic carbon or natural variability.

**Figure 5:**
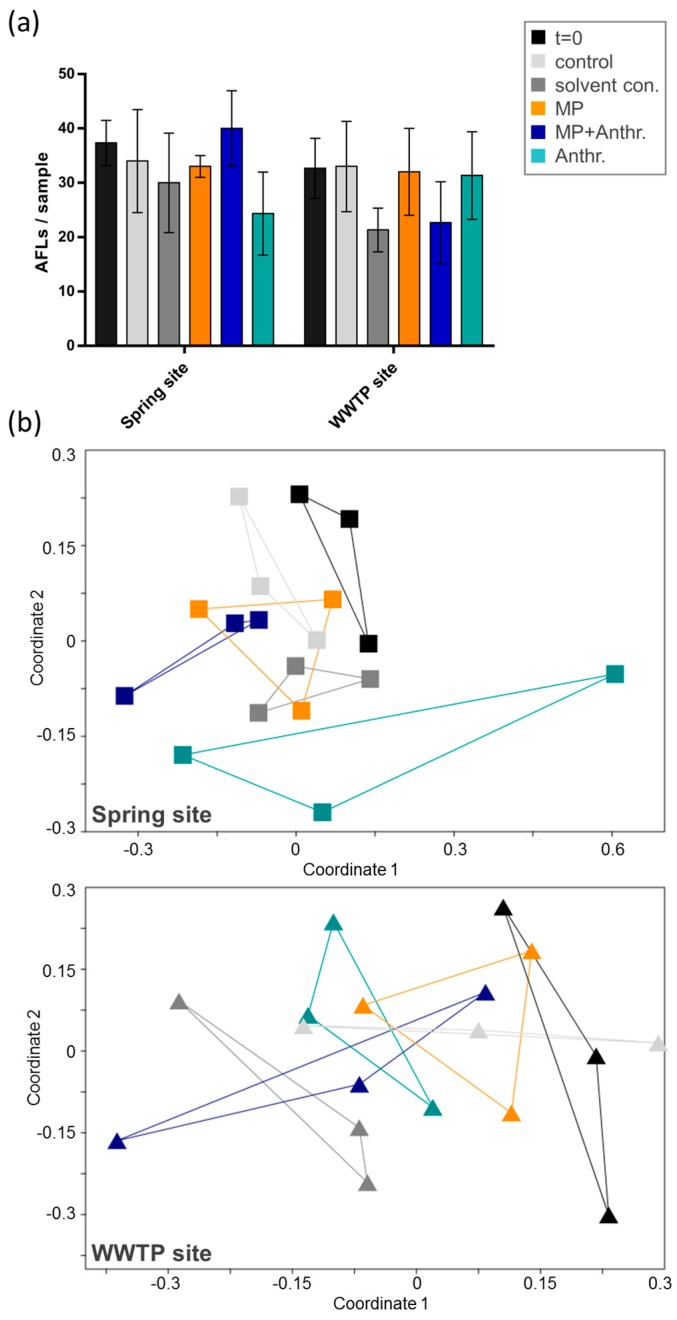
(**a**) Number of Automated Ribosomal Intergenic Spacer Analysis Fragment Length (AFLs) as indicator of bacterial richness in sediments from a spring site and downstream of a wastewater treatment plant (WWTP) before and after treatment with anthracene (Anthr.), polyethylene particles (MP) and anthracene-loaded polyethylene particles (MP + Anthr.). (**b**) The data set from (**a**) is represented in an nMDS graph based on Bray-Curtis similarities between samples, showing the shift in bacterial communities before and after treatment.

The anthracene did not induce a significant shift in the bacterial composition (Figure 5) and the bacterial diversity (AFL numbers) remained unchanged. In the sediment from the spring site the treatment with anthracene without PE particles induced a slight, albeit overall not significant (R < 0.19, Table S6, one-way ANOSIM), shift in the bacterial composition, whereas all other treatments were similar to the control (R < 0.03). The generally weaker reaction to anthracene may also stem from low fluorescent signal intensity in the fragment length analysis for this experiment.

**Figure 6:**
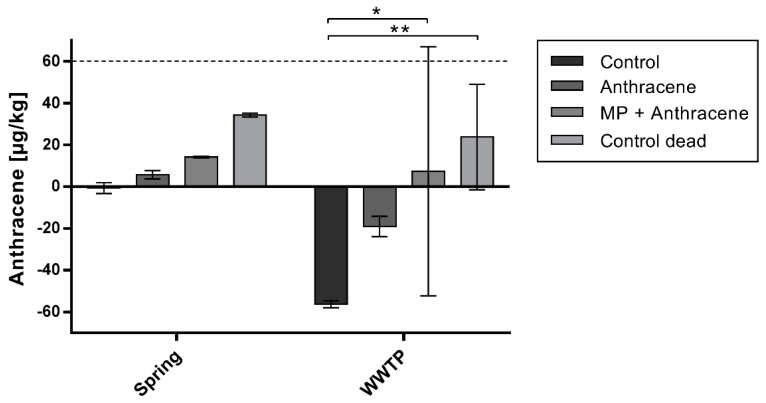
Anthracene concentrations detected in the sediment after two weeks of incubation, minus the respective ambient background concentration of anthracene detected in the environmental sample. The spiked concentration of anthracene is displayed in the dashed line (60 µg/kg). Significant differences (two-way ANOVA; * *p* < 0.05, ** *p* < 0.01) were detected between the control group and the treatment with microplastic (MP) + Anthracene and the dead control. Even though high amounts of sodium azide were added to the dead control, anthracene was still degraded. Different treatments at the spring site were not significant using the two-way ANOVA due to the high variance at the wastewater treatment plant (WWTP).

In contrast to the bacterial community composition, the measurement of anthracene concentrations in the sediment after two weeks of incubation did show a significant impact on bacterial degradation by different treatments (Figure 6). Significantly less anthracene was degraded in the presence of MP than without MP for sediment from the WWTP (two-way ANOVA). The same trend was observed for sediment from the spring but high variations in the data from the WWTP prevented significance using the two-way ANOVA. Using the one-way ANOVA and the Bonferroni post-test on the spring dataset, significant differences (*p* < 0.05–*p* < 0.0001) between all groups were observed (Table S7). Degradation rates of anthracene were higher in the sediment collected downstream of the WWTP than at the spring site. In the control of the latter, no degradation of the ambient background anthracene concentration took place, whereas at the WWTP 56 µg/kg anthracene was reduced. The addition of sodium azide inhibited but did not stop bacterial degradation completely. In the water phase, anthracene was below the detection limit (0.01 µg/L) as it was suggested by calculations on equilibrium distribution. Also, phenanthrene present in the ambient background of the sediment was degraded in the experiment spiked with anthracene (Figure S1 in Supplementary file). Degradation was higher in the sediment from downstream of the WWTP than from the spring. The ambient background concentrations in the sediment were as follows: phenanthrene: 109 µg/kg (at the spring), 1160 µg/kg (downstream of the WWTP); anthracene: 15 µg/kg (at the spring), 154 µg/kg (downstream of the WWTP).

## 4. Discussion

Even though freshwater sediments may represent a major sink for both MPs and associated pollutants, the environmental effects of MPs on these sediments are understudied to date. The accumulation of these compounds in sediments might have significant consequences regarding the structure and function of these habitats [52,53]. Here we present first evidence that MPs can affect microbial species numbers and community composition in freshwater sediments. The effects of MPs on natural sediment bacteria were only visible in sediment from the river spring, a relatively unpolluted site. In contrast, an effect on sediment from a polluted environment, i.e., after the wastewater treatment plant effluent, was not observed. The latter suggests that pristine environments are more sensitive to microplastic contamination, whereas bacteria downstream of the wastewater treatment plant effluent may be more adapted to physical and chemical stressors [39,42].

The microplastic concentrations used in our study were about three to four orders of magnitude higher (2–20 mg MP/g sediment) compared to what has been detected in natural freshwater sediments (4.3 × 10^−4^ to 7.1 × 10^−3^ mg MP/g sediment, quantified by Klein et al. [9] and calculated from given particle numbers assuming a diameter comparable to our particles). Currently reported amounts of MPs in the environment are therefore unlikely to change microbial communities in a time span of 14 days. Nevertheless, MP contamination in the environment may be underestimated [54] and the amounts of MPs used in this study may be realistic at pollution hotspots, or, with an increasing input of MPs to the environment, may become more realistic in the future. Moreover, short-term laboratory experiments with high concentrations may resemble long-term effects in environments with lower concentrations. MPs accumulating in lower concentrations over months and years may therefore affect bacterial communities in natural sediments.

The underlying mechanisms for the observed changes in the microbial communities remain unclear, however, physical as well as chemical changes induced by MPs are likely. The surface of MP particles represents a new habitat for bacterial growth, whereby certain taxa are expected to thrive and, as a result, the composition of bacterial communities in the sediment may change [55]. Moreover, MPs can change the abiotic properties in sediments like porosity, which might also affect bacterial communities [56]. MP particles as a new phase in the water-sediment system also change concentration equilibria of chemical compounds [32]. Plastic additives, such as phthalates may leach from the microplastic matrix especially shortly after production. This was not the case for the material used in this study but may be the case for other types of plastic. The leached compounds can be harmful to some micro- and macroorganisms [57] but can also serve as carbon source for certain types of bacteria [58].

In line with the latter, it has been speculated that MPs may also serve as a transport vector for chemical pollutants, including hydrophobic compounds such as PAHs [29,59]. In this study, we tested the transport capacity of PE particles for two PAHs, phenanthrene and anthracene. In the experiment, the total transported mass of phenanthrene and anthracene per gram sediment was in the range of ambient sediment concentrations in polluted environments [46]. Phenanthrene increased the ambient background concentrations in the Ammer river sediment by 46% at the spring and by 14% downstream of the WWTP and anthracene by 390% and 49%, respectively. Even though the concentrations of phenanthrene and anthracene in the spiking solution were very high and above environmentally relevant concentrations, as was the amount of PE particles used for exposure of sediment (see above), the transport potential of MP particles for organic pollutants was confirmed by observations in this study.

In contrast, natural and anthropogenic compounds from the ambient chemical background of the sediment may sorb to MP until equilibrium is reached, thereby reducing the ambient background concentration of these compounds in the water and sediment phase. Also in our experiments “clean” PE particles may have sorbed some of the background chemicals from the original sediment. This may change biodegradation rates but also toxicities, depending on the type of compound and the studied organism. However, to date it is unclear, how and if chemicals loaded on MP particles are bioavailable for organismal processes [34].

In our experiments, the addition of two PAHs, anthracene and phenanthrene, without MP induced changes in the bacterial community composition and degradation. Most likely the compounds induced a shift towards bacteria benefiting from the presence of the organic compounds (and the solvent acetonitrile for phenanthrene) as a carbon source [41]. Due to the high ambient background of both PAHs especially at the site downstream of the WWTP, sediment bacteria from this environment may be adapted to the presence and degradation of PAHs. This is supported by higher degradation rates of anthracene and phenanthrene in sediment from downstream of the WWTP and a weaker reaction in bacteria community shifts compared to sediment from the spring, where ambient PAH concentrations are about ten times lower. Bacterial degradation of phenanthrene in natural river sediment over a 14 days incubation period has been described previously by Verrhiest et al. [41]. The shifts in the bacterial communities as a response to the treatment with both PAHs may also be explained by a toxic effect of the compounds. However, since the species richness was not reduced in the presence of phenanthrene and anthracene, a toxic effect does not seem likely. Moreover, it was reported that anthracene, phenanthrene and acetonitrile have no toxic effect on microorganisms even at much higher concentrations then used in this study [41,60].

Calculations on equilibrium distribution suggest that in a system without PE particles, freely dissolved phenanthrene and anthracene would be absorbed by the sediment particles. Since the sorption capacity of PE particles for phenanthrene and anthracene is similar to that of natural sediment particles [61], similar effects should be expected in the presence of MPs. Nevertheless, in the present study the same total amount of each compound sorbed to PE particles induced a different response in natural river sediment bacterial community composition compared to the compound without MPs. Moreover, we observed decreased microbial degradation of anthracene in the presence of MPs. This suggests that phenanthrene and anthracene, when absorbed by MP, are not or less bioavailable than when absorbed by natural sediment particles. An explanation might be, that the fraction which is transferred between the particulate phases will stay shortly in the water phase and hence may be subject to biodegradation before sorbing to the sediment. Another explanation for the decreased bioavailability might be that natural sediment particles are covered with a microbial biofilm which can biodegrade the organic compounds. Biofilm formation of various bacteria on activated charcoal has been shown to enhance degradation of phenanthrene [62]. The PAHs, instead of moving into the sediment particle may thereby be trapped in the biofilm, where they may be available for various microbiological processes [63]. MP particles in the experiment were initially not covered by a biofilm. Compounds sorbed to the plastic may therefore be unavailable for microbial degradation.

Similar results were derived in a study by Beckingham and Ghosh [61] on the bioavailability of polychlorinated biphenyls (PCBs) for sediment worms in the presence of MP particles. The latter study found that PCBs were absorbed better or in the same range by PE particles and natural organic material, however the bioavailability of PCBs for the worms was reduced when absorbed by PE particles. In a multimedia modelling approach, Gouin et al. supported the fact that the uptake of chemicals into organisms can be neglected compared to natural sources [64]. The same was experimentally shown by Rehse et al. in this issue [36].

## 5. Conclusions

In this study we showed, that MP particles (polyethylene) have the potential, to alter sediment bacterial community composition albeit in high concentrations (20 mg/g sediment) and in relatively unpolluted river sections, whereas locations influenced by WWTP effluent were insensitive towards MPs. Long-term exposure to higher future levels of MPs may influence bacterial communities and, in this way, basic biogeochemical processes in the aquatic (and possibly also terrestrial) ecosystem.

Due to high ambient concentrations of organic pollutants and their sorption to natural particles, the transported amounts of two PAHs (anthracene and phenanthrene) did not add substantial quantities to background environmental levels in the sediment. Thus, the current environmental concentration of MP-transported hydrophobic organic pollutants is not expected to have a major effect on sediment organisms, especially at anthropogenically influenced sites. Nevertheless, the presence of MP reduced the effect of the two PAHs on microbial community composition and the degradation of these organic compounds. This was especially the case in sediments located downstream to the effluent of a WWTP, where degradation rates of PAHs were one to two orders of magnitude higher than in sediments from the river spring. If the presence of MP particles in wastewater treatment plants or at other highly polluted sites reduces bacterial degradation of hydrophobic chemicals, the mobility of chemical compounds may be altered. Chemicals absorbed to MPs may be transported further and into pristine ecosystems, where they may desorb from the particles. In this way residence times of pollutants may be elongated by the presence of MPs, which could lead to slow accumulation and to long-term chronic exposure of micro- and macroorganisms.

## Figures and Tables

**Table 1:** Mass of each phase in the batch, their respective organic carbon concentrations (c_OC_) and the calculated fraction of phenanthrene (f_Phen_) and anthracene (f_Anth_) in each phase after equilibration.

	Microplastics	Sediment	Pore Water	Water
**Spring**				
Mass [g]	0.02	5.5	4.5	20
c_OC_	-	18.7 g/kg dw	8.7 mg/L	2.4 mg/L
f_Phen_ [%]	15.4	83.4	0.3	1.0
f_Anth_ [%]	15.6	83.1	0.3	1.0
**WWTP**				
Mass [g]	0.02	5.2	4.8	20
c_OC_	-	22.5 g/kg dw	9.0 mg/L	5.4 mg/L
f_Phen_ [%]	13.8	85.0	0.2	0.9
f_Anth_ [%]	14.0	84.8	0.2	1.0

## Supplementary Materials

The following are available online at http://www.mdpi.com/1660-4601/15/2/287/s1, Figure S1: Degradation of phenanthrene, Table S1: ANOSIM R Values of the treatment with microplastic of sediment collected downstream of a WWTP, Table S2: ANOSIM R Values of the treatment with microplastic of sediment collected at the river spring, Table S3: ANOSIM R Values of the treatment with phenanthrene and microplastic of sediment collected downstream of a WWTP, Table S4: ANOSIM R Values of the treatment with phenanthrene and microplastic of sediment collected at the river spring, Table S5: ANOSIM R Values of the treatment with anthracene and microplastic of sediment collected downstream of a WWT, Table S6: ANOSIM R Values of the treatment with anthracene and microplastic of sediment collected at the river spring, Table S7: Bonferroni multiple comparison post-test on the one-way ANOVA for differences between treatments in anthracene concentrations of the spring sediment.
