# Supplementary material for: Microplastics Reduce Short-Term Effects of Environmental Contaminants. Part II: Polyethylene Particles Decrease the Effect of Polycyclic Aromatic Hydrocarbons on Microorganisms
Source: Int J Environ Res Public Health. 2018 Feb 7;15(2):287. doi: 10.3390/ijerph15020287 (PMC5858356; doi:10.3390/ijerph15020287)
Supplement: Supplementary file 1 [file ijerph-15-00287-s001.pdf]

Supplements

Microplastics reduce short-term effects of environmental contaminants.

Part II: Polyethylene particles decrease the effect of polycyclic aromatic hydrocarbons on microorganisms

Julia Kleinteich, Sven Seidensticker, Nikolaj Marggrander, Christiane Zarfl

Center for Applied Geoscience, Eberhard Karls Universität Tübingen, D-72074 Tübingen, Germany

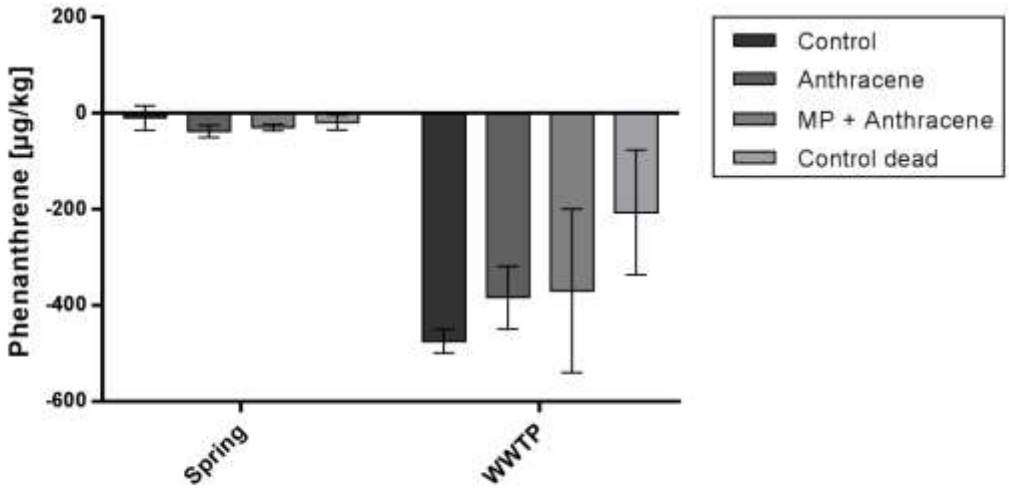

Figure S1: Degradation of phenanthrene measured in the sediment after two weeks of incubation with spiked anthracene and/or microplastic (MP) particles, minus the ambient background concentration of phenanthrene (109 µg/kg at the spring and 1160 µg/kg at the wastewater treatment plant - WWTP).

Table S1: ANOSIM R Values of the treatment with microplastic (MP) of sediment collected downstream of a wastewater treatment plant.

	Control	*MP low*	*MP high*
Control		-0.15	0
MP	-0.15		-0.22
MP high	0	-0.22	

Table S2: ANOSIM R Values of the treatment with microplastic (MP) of sediment collected at the river spring.

	Control	*MP low*	*MP high*
Control		0.41	0.93
MP	0.41		0.20
MP high	0.93	0.20	

Table S3: ANOSIM R Values of the treatment with phenanthrene (Phen) and microplastic (MP) of sediment collected downstream of a wastewater treatment plant.

	Control	*MP*	*MP+Phen*	*Phen*
Control		0.30	0.57	0.74
MP	0.30		0.09	0.69
MP+Phen	0.57	0.09		0.85
Phen	0.74	0.69	0.85	

Table S4: ANOSIM R Values of the treatment with phenanthrene (Phen) and microplastic (MP) of sediment collected at the river spring.

	Control	*MP*	*MP+Phen*	*Phen*
Control		0.28	0.59	0.26
MP	0.28		0.37	0.70
MP+Phen	0.59	0.37		0.44
Phen	0.26	0.70	0.44	

Table S5: ANOSIM R Values of the treatment with anthracene (Anthr) and microplastic (MP) of sediment collected downstream of a WWTP.

	Control	*Solv. control*	*MP*	*MP+Anthr*	*Anthr*
Control		0.0	0.09	0.11	0.0
Solv. control	0.0		0.13	-0.37	-0.04
MP	0.09	0.13		0.17	-0.22
MP+Anthr	0.11	-0.37	0.17		0.0
Anthr	0.0	-0.04	-0.22	0.0	

Table S6: ANOSIM R Values of the treatment with anthracene (Anthr) and microplastic (MP) of sediment collected at the river spring.

	Control	*Solv. control*	*MP*	*MP+Anthr*	*Anthr*
Control		0.07	-0.19	-0.15	0.31
Solv. control	0.07		-0.22	0.30	0.0
MP	-0.19	-0.22		0.33	0.11
MP+Anthr	-0.15	0.30	0.33		0.33
Anthr	0.31	0.0	0.11	0.33	

Table S7: Result of the Bonferroni multiple comparison post-test on the one-way ANOVA for differences between treatments in anthracene concentrations after two weeks of incubation of spring sediment.

Treatment	***Mean Diff***	***95.00% CI of diff.***	***Significant?***	***Summary***	***Adjusted P Value***
Control vs. Anthracene	-6.408	-11.31 to -1.504	Yes	*	0.0113
Control vs. MP + Anthracene	-14.87	-19.77 to -9.964	Yes	****	<0.0001
Control vs. Control dead	-34.93	-39.83 to -30.02	Yes	****	<0.0001
Anthracene vs. MP + Anthracene	-8.46	-13.36 to -3.556	Yes	**	0.0019
Anthracene vs. Control dead	-28.52	-33.42 to -23.61	Yes	****	<0.0001
MP + Anthracene vs. Control dead	-20.06	-24.96 to -15.15	Yes	****	<0.0001
